# Why should we strive to let them thrive? Exploring the links between homecare professionals thriving at work, employee ambidexterity, and innovative behavior

**DOI:** 10.1186/s12913-025-12293-9

**Published:** 2025-01-27

**Authors:** Terje Slåtten, Barbara Rebecca Mutonyi, Gudbrand Lien

**Affiliations:** 1 Inland School of Business and Social Science, University of Inland Norway, Campus Lillehammer, 2604 Lillehammer, Norway; 2 Kristiania University College, School of Economics, Innovation and Technology, 1153 Oslo, Norway

**Keywords:** Thriving at work, Employee ambidexterity, Exploration, Exploitation, Innovative behavior, Homecare professionals, Homecare service

## Abstract

**Background:**

The concept of thriving at work (TAW) has received increased interest within health services research in recent years. TAW embraces employees’ experience of being energized and feeling alive when employed in an organization. However, previous research has been limited mainly to the investigation of factors that promote TAW. Consequently, there is a lack of research linking TAW to potential outcomes. Based on this knowledge gap, this study aimed to examine links between TAW and two potential outcomes: employees’ individual innovative behavior (IIB) and employee ambidexterity (EA). Thus, the study contributes to a relatively neglected area, homecare, within the domain of health services research.

**Methods:**

In this cross-sectional study, *N* = 258 Norwegian homecare professionals in nine municipalities were selected through convenience sampling. The conceptual model's results were analyzed using partial least-squares structural equation modeling with SmartPLS 3 software. The study tested both direct and indirect relationships. Indirect relationships were achieved through bootstrap.

**Results:**

The main results from the empirical study can be summarized as follows: (i) TAW was found to be positively linked to both EA (*b* = 0.46) and IIB (*b* = 0.22); (ii) TAW and EA explained about 30% (*R*2 = 0.29) the variance in IIB; (iii) The relationship between TAW and IIB was found to be mediated by the EA; (iv) TAW was positively linked to each of the two dimensions that constitute EA. However, when comparing the individual strength of linkages, TAW was found to be most strongly linked to the exploitation dimension of EA (*b* = 0.50) and less strongly linked to the exploration dimension of EA (*b* = 0.35).

**Conclusions:**

Employees’ level of TAW in homecare services is linked to desirable outcomes, as represented by EA and IIB in this study. Managers should be aware of the development and changes in their employees' TAW levels. Consequently, continuously monitoring and cultivating the TAW of individual employees to determine whether they experience a sense of being energized and feeling alive as members of the organization is an important practical implication. TAW is a key to essential outcomes. Managers should, therefore, strive to let all of their employees thrive.

**Supplementary Information:**

The online version contains supplementary material available at 10.1186/s12913-025-12293-9.

## Background

The Greek philosopher Aristotle’s quote, “*The final end of human life is to flourish, to live well, to have a good life”* [1], posits that the actions and purpose of human life, in one way or another, are the means for fulfilling an ultimate wish or goal, in order to “flourish … to have a good life”. To Aristotle, this reasoning is logical because he believed few humans would really want to have a poor life [1].

Many domains of a human’s life act as the potential means for achieving Aristotle’s “good life” [1]. One such domain is the work life of humans. Considering all the hours of the day, the energy, and the physical and mental self-sacrifice devoted to one’s employer, it is reasonable to assume that there is a natural desire to experience a “good life” at work and to have the perception of thriving as a member of an organization.

Considering its importance, this paper focused on homecare employees’ experience of having a good work-life, specifically by adopting the relatively new concept labeled thriving at work (TAW). TAW was first proposed by Spreitzer et al. in 2005 [2]. The concept reflects employees’ experience of being energized and feeling alive when employed in an organization [3]. TAW has been considered an essential aspect of employment, work, and quality of life [4]. The concept has received increased interest and awareness because TAW is critical for employees [5, 6]. Regarding the importance of TAW, Walumbwa et al. state: “In today’s fast-growing and competitive knowledge-based service economy, a thriving workforce is essential for an organization’s competitive advantage and sustainable performance” [5].

The concept of TAW has received increased attention. However, previous research has been undertaken mainly within private organizations. A consequence of this one-sided focus has resulted in scarce research on TAW within public organizations. More research needs to be done on the TAW of employees within public healthcare services, which is surprising because healthcare services are a significant part of the knowledge-based service economy, in which human resources are considered essential to the quality of services offered to customers. Consequently, homecare professionals, the target group of this study, are relevant and essential employees to include in studies of TAW.

The very few previous studies on TAW within healthcare settings have most often focused on the precursors to TAW. Examples of precursors include psychological capital and perceived organizational support [7], self-empowerment [8], autonomy [9], job satisfaction [10], self-efficacy [11], and job resources and job attachment [12]. Because of this one-sided focus, we know very little about the potential outcomes of TAW. To our knowledge, only one scientific work has identified potential outcomes of TAW within a healthcare context. Moloney et al. [13] suggested the sustainability of human resources and better health and well-being to be two potential outcomes of TAW. However, their study was limited to linking TAW conceptually to potential outcomes. Consequently, there is a significant need for more empirical research focusing on potential outcomes linked to TAW within a healthcare setting.

Based on the knowledge gap regarding TAW in previous research, this paper aimed to examine the link between health professional TAW and two specific outcomes of TAW: employees’ innovative behavior and employees’ ambidexterity. There are three reasons for this choice of outcomes. First, there is a lack of studies focusing on both innovative behavior and ambidexterity [14]. Second, recently, there has been an explicit call for more research on employees’ innovative behavior, ambidexterity, and TAW within healthcare services [14]. Third, Walumbwa et al. claim that “we do not yet understand the process through which thriving relates … to outcomes” [5] and suggest ambidexterity (studied on an individual level) to be a process variable between TAW and employees’ innovative behavior that may address this knowledge gap. We aim to address the knowledge gap and the call for more research on these concepts. To our knowledge, this is a pioneering study that examines the links between the three suggested concepts within a healthcare context. Our paper offers new knowledge and insight and thus contributes to an important area within the domain of health services research.

### Conceptual model

**Fig. 1:**
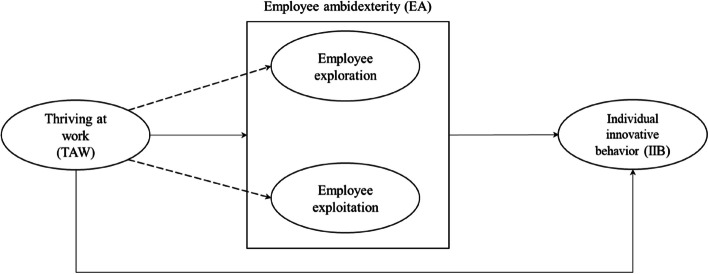
Conceptual model

Figure 1 presents the conceptual model to be empirically tested. As shown, it is assumed that TAW is linked directly to both individual innovative behavior (IIB) and employee ambidexterity (EA).

In addition, the box in the middle of the figure suggests that EA functions as a mediator in the relationship between TAW and IIB. The broken lines indicate that TAW is assumed to be linked directly to each of the two dimensions that constitute EA (employee exploration and employee exploitation). The following section will elaborate in detail on the proposed conceptual model (Fig. 1) and its hypotheses.

### Review of the literature

This section first explains and defines the TAW concept before discussing the proposed links between TAW and employees’ IIB and EA, as visualized in the conceptual model in Fig. 1.

### Thriving at work (TAW)

In line with previous research, thriving at work (TAW) is defined in this study as an employee’s psychological state composed of the joint experience of vitality and learning [2, 15]. There are several essential aspects to this definition. First, thriving is a pleasant subjective experience of an individual person. TAW is reflected in the individual’s positive feelings, including enhanced insight, knowledge, and perception of vitality and aliveness, and making progress in one’s self-development [2].

The first of the two TAW dimensions, *vitality*, is portrayed by Spreitzer et al. as a person’s positive sense of having accessible energy and feeling alive. The second dimension, *learning,* embraces a person’s sense of continually acquiring valuable knowledge and skills and the positive experience of gaining capability in applying them [2]. The dimensions reflect two aspects of a person’s psychological experiences: the affective aspect of development (vitality) and the cognitive aspect of making progress (learning).

Furthermore, the two dimensions of thriving reflect two perspectives of individuals' development and psychological functioning: the hedonic (vitality) and the eudaemonic (learning). The hedonic perspective, captured in the vitality dimension of thriving, refers to individuals pursuing enjoyable experiences. In contrast, the eudaemonic perspective, depicted in the learning dimension of thriving, refers to individuals having an inherent desire to realize and achieve their maximum potential as human beings [2].

Although the concept of thriving consists of two dimensions, an important and fundamental premise of the concept of TAW is the “joint experience” that really constitutes the construct [2, 15]. The TAW concept is studied as a joint experience because the two dimensions (vitality and learning) are, based on psychological experiences, interconnected with each other [6]. This is in line with Porath et al., who remarked that “although each dimension can signify some progress toward growth and personal development at work, it is only in concert that they enhance one another to form the experience of thriving” [15].

A final aspect that characterizes TAW is that it is defined as a “psychological state”. Defining the concept as a “state” rather than a “trait” of an employee means that TAW is not considered to be a relatively immutable part of the personality or enduring disposition of a person. Defining TAW as a psychological “state” infers what Spreitzer et al. describe as a “temporary internal property of an individual” [2]. This means that an employee’s experience of TAW is relatively short-term dynamic, and has the potential for positive or negative change over time. TAW is also affected by employees' work context [2]. To study TAW as a “state” of a person is something many find interesting because it implies that TAW is manageable. Consequently, there is potential for managers and leaders of organizations (e.g., within the domain of healthcare organizations) to take either strategic or practical steps to positively influence, cultivate, and change the TAW of employees in a desired direction.

TAW has received much attention in recent years and has been described in favorable terms, such as “extremely important” [16], “essential for an organization” [5], and a “key factor in improving performance” [16].

It is reasonable to assume that employees’ TAW is positively associated with several desirable outcomes for organizations. Figure 1 illustrates the two such outcomes included in this study. These concepts—innovative behavior (IIB) and employee ambidexterity (EA)—and their linkages with TAW are elaborated in the following.

### Individual innovative behavior (IIB)

Glover et al. [17] describe our present time as “an innovation age for healthcare delivery.” Much time, effort, and money are invested in supporting innovation in healthcare organizations [17]. Innovation can be manifested in countless ways in an organization, including, for example, new products and services, new service processes, new structures, and new administration systems [18], and not limited to a specific place, position, department, person, or group within an organization.

Due to the broad spectrum of approaches to innovation, innovation has been described as a “difficult phenomenon to define” [19]. The study object for innovation in this study is limited to the individual homecare employees in the health organizations. Specifically, it focuses on those employees who work at the front in homecare service organizations that offer health services to their customers. We refer to these employees as homecare professionals and are interested in whether innovation is present and manifested in their work role behavior.

In line with previous research on innovation in individual employees, individual innovative behavior (IIB) refers to [homecare professionals’] intentional creation, introduction, and application of new ideas within a work role to benefit [their] role performance [20]. In this definition, IIB is related to the health professional’s specific work role and reflects new ways for homecare professionals to perform their daily work. It is notable that IIB does not distinguish between types of innovative behavior manifested in the health professional’s role performance, which may range from incremental innovation at one end of the continuum to radical innovation at the other. Thus, IIB encompasses only the existence of potential new change-oriented activity [21] among homecare professionals independent of the type of change (incremental versus radical).

As shown in Fig. 1, there is an assumption that IIB and TAW are linked. TAW is, as defined in the previous discussion, a psychological state of joint experience of learning and vitality [2, 15]. According to Abib et al., when TAW is present it is capable of transforming individuals to yield positive behavioral outcomes [22].

Previous research has considered TAW to be capable of promoting a variety of desirable (behavioral) outcomes in organizations. For example, TAW has been found to be positively related to employees’ attitudes toward self-development [23], organizational commitment [24], task performance [25], and creative performance [6, 26]. Based on the two characteristics that constitute TAW (referring to vitality and learning), it is reasonable to assume that TAW is capable of functioning as a psychological momentum to actually perform the IIB of employees. This idea is supported by Carmeli and Spreitzer, who state that thriving captures the central tenant of motivation as it reflects an energetic force (i.e., vitality) that can direct work-related behavior [27]. It is reasonable to assume that one relevant example of such work-related behavior could be the IIB of employees.

In this study, IIB is understood as what is often labeled an extra-role behavior. As such, its performance may depend on the employee’s perception of work-related circumstances and experience of being a member of the organization (e.g., whether they are thriving or not). Employees whose daily work is in the front of the health organization (which is the case of participants in this study) have a unique opportunity to observe and learn from their daily work role experiences. Through these experiences, they can identify areas where there is a potential for positive change and improvement in their work role. Areas for positive change could include identifying potential improvements in service processes, new service procedures, and new ways to offer parts of the total “service package.” Those employees who possess TAW will not be limited to stagnation and observing “gaps” where positive change is required. Instead, individual employees with TAW who embrace the joint experience of learning and vitality [2] will be motivated and actively engaged to close these gaps. This voluntary engagement and willingness to close the gaps is manifested through their IIB. Consequently, TAW will energize and positively serve as fuel for the IIB of employees [27].

Previous research, although undertaken outside healthcare services, supports a direct positive relationship between TAW and employees’ IIB [22, 27, 28]. Therefore, we expected to find the same relationship pattern between TAW and IIB among homecare professionals. This reasoning leads to the following first hypothesis in this study:

#### Hypothesis 1:

There is a positive relationship between TAW and their IIB.

### Employee ambidexterity (EA)

Employee ambidexterity (EA) was placed in the center of Fig. 1. This placement has three indications. First, EA is expected to be linked to both TAW and IIB individually. Second, it signals that EA bridges or, in more formal terms, operates as a mediator between TAW and IIB. Third, placing EA between TAW and IIB means that EA represents and captures a process embedded in individual employees. Consequently, EA is assumed to have a central role.

According to the Merriam-Webster Dictionary, the meaning and content of the word “ambidexterity” can be understood as using both hands with equal ease or dexterity [29]. Ambidexterity refers to the capability to combine two things, a duality, that seems at first glance to be extremely difficult or impossible to achieve or perform simultaneously.

The literature shows that ambidexterity has been studied in a variety of ways and from different perspectives, including leadership [30], manager [31], team [32], individual [30, 33], and organization [34]. There is a range of opportunities for studying ambidexterity in organizations. However, this study has focused on EA at the individual level. There are three reasons why this study focuses on EA at the individual level. First, there is a major lack of research studies focusing on ambidexterity at an individual level [30, 33]. Recently, there have been explicit calls for more research on ambidexterity from this perspective [34, 35]. Second, previous research has suggested that individual EA is a vital premise and a prerequisite for actually achieving other types of ambidexterity, such as organizational ambidexterity [36]. Thus, EA is of fundamental importance. Third, because the other two concepts of TAW and IIB take an individual perspective, it appeared logical that EA should be studied in the same way. Consequently, the three concepts included in the study (TAW, EA, and IIB) are examined at the individual level of perspective.

EA refers to the combination of two contradictory activities: exploration activities and exploitation activities. When it does so, it seeks to adjust and implement March’s conceptualization of the two types of activities in such a way to become appropriate and adoptable to the study object of this specific study, namely homecare professional within health care services [37].

March described and exemplified exploration activities as “things captured by terms such as search, variation, risk-taking, experimentation, play, flexibility, discovery” [37]. Exploitation activities, in contrast, represent “refinement, choice, production, efficiency, selection, implementation, execution” [37]. While the exploration activities are potentially divergent, with the aim of creating changes and variation in one’s experience [31] when seeking new input, broadening and stretching one’s present learning and knowledge bases, exploitation activities are potentially convergent, with a narrow variation of one’s practices and seek consistency in one’s experience [31]. Consequently, exploitation activities seek to deepen and refine one’s existing insight and utilize one’s present base of knowledge [31]. Thus, the adoption of EA in this study is about homecare professionals’ devotion to exploration and the exploration embedded in their work roles and practices.

There are two essential aspects of the abovementioned definition of EA to bear in mind in this study. First, the concept of EA is defined as an “activity,” indicating that the concept does not reflect an inherent, relatively intangible psychological trait of a person. EA is a behavioral construct, a relatively tangible and observable capability of a person. Second, EA consists of two dimensions [37]. Although exploration and exploitation activities are separate and contradictory, it is the sum of the two dimensions that actually constitutes EA. Consequently, this study takes a compensatory interpretation and understanding of EA. It is reasonable to assume that a person will be more oriented toward one of the dimensions of EA (e.g., prefer exploitation activities to exploration activities). It is assumed that in relatively few situations, there will be an exact balance between the two “ingredients” in EA; most likely, one dimension of EA will dominate over the other. Based on a compensatory interpretation of EA, high levels of EA will most likely occur when both dimensions of EA are high. Therefore, EA in organizations will not be homogenously distributed across employees, but it will vary from one individual employee to another. The variation in the level of EA among employees is dependent on how it is linked to positive and negative factors.

This study aimed to determine whether EA is linked to individual employees’ TAW. The basic premise for assuming the existence of a linkage between TAW and EA is that human behavior is always caused or triggered by something or someone. There is always a reason as to why a behavior occurs. The source that affects or drives EA in this study is the TAW of individuals. This assumed link is supported by previous research on TAW. Specifically, TAW has been described in the literature as a “critical psychological driver of individual growth and development” [38]. Following this line of reasoning, individual employees who positively experience TAW, a psychological state [2], will be motivated to search for sources and engage in activities related to EA. Because of TAW, employees will involve themselves in activities that provide them with access to new sources of knowledge, new experiences that could potentially be useful for their work role (exploration activities), and activities that contribute to deepening their existing knowledge and experience (exploitation activities), thus refining and perfecting their work role and practices. According to Spreitzer et al.’s original work [2], there is a mutual linkage between employees’ experience of TAW (a psychological state) and their (behavioral) work-related activities (referred to as EA in this study). This mutual and positive linkage is claimed to form and constitute a “self-sustaining … positive spiral occurring within individuals” [38]. Based on these ideas, TAW and EA will, therefore, have a positive strengthening and self-reinforcing impact on each other and, further, will form a positive upward spiral, serving as fuel for each other. To our knowledge, no previous research study has examined the link between TAW and EA. However, previous research has found a positive relationship between TAW and concepts associated with ideas embraced in the concept of EA, such as extra-role behavior [39], taking proactivity/charge [40], and in-role work behavior [5]. Consequently, there are good reasons to expect to find a positive relationship between TAW and EA. This reasoning leads to the following second hypothesis in this study:

#### Hypothesis 2:

There is a positive relationship between TAW and EA.

Building on H1 and H2, it is suggested that TAW is linked to IIB through a mediating process manifested by EA. Previous research seems to have only studied the direct positive relationship between TAW and IIB (e.g., [22, 27, 28]). Consequently, EA as a mediator represents an alternative route to TAW’s effect on IIB. It is assumed that, by including EA as a mediator, a more complete and realistic description of the underlying construct and process will be obtained, thus explaining why a relationship exists between TAW and IIB. It is supposed that the exploration and exploitation activities involved in EA are a necessary process and thus function as a natural bridge between TAW and IIB.

The basic premise is that before IIB can be manifested, there must be two preconditions in place. First, IIB (incremental or radical) needs certain input before its behavioral manifestation in a specific work role. This means that employees must have a behavioral orientation toward combining the refinement of their existing knowledge and/or experience and creating new knowledge and/or experiences. This behavioral orientation serves as a necessary input to IIB and is captured by the process embedded by EA. However, this is not sufficient. There must also be another precondition of fundamental importance in place; employees must also possess a certain level of motivational resource that is strong enough and qualifies to drive both EA and IIB. In this study, this fundamental driving capability stems from TAW.

Previous research has described TAW as a “critical psychological driver of individual growth and development … and … essential for … performance [38]. Consequently, based on this description, TAW (psychological driver), EA (growth and development), and IIB (performance) can be argued to be highly linked in forming a logical chain of reactions beginning with an initial triggering factor (employees’ TAW) to start the domino effect. Specifically, previous studies by Usman et al. (2020) explored the links between ambidextrous leadership, innovative work behavior, and workplace thriving and found positive and significant relationships. To the best of the authors´ knowledge, this study is a pioneer in health services research that explores the proposed links between TAW, IIB, and EA. Therefore, though the study by Usman et al. (2020) is not similar to this study, it provides an indication that there are positive links to be expected. As such, this study assumes that a positive shift in EA, stemming from employees’ TAW, will next serve as a (necessary) ingredient or input, leading to an increase in IIB. Consequently, EA is expected to operate as a mediator between TAW and IIB. This thinking leads to the following third hypothesis:

#### Hypothesis 3:

The relationship between TAW and IIB is mediated by EA.

Consistent with previous research, EA is studied here as a concept consisting of two dimensions (exploratory and exploitive behavioral activities). Based on the compensatory interpretation of EA, the two dimensions are combined into one. In this fourth and final hypothesis, we break away from this pattern of studying EA. We are still interested in examining the links between TAW and EA [Hypothesis 2]. However, in contrast to previous studies that merge the two dimensions of EA into one, we are interested in exploring the links between TAW and each individual dimension of EA separately. This approach represents an exploratory part of this paper. In Fig. 1, this is shown by the use of a dotted line between TAW and the two dimensions that constitute EA.

As mentioned previously, EA is a combination and mixture of both exploratory and exploitative activities. This approach provides a more nuanced and detailed insight than the compensatory approach in two ways. First, it reveals how much impact TAW has on each individual dimension of EA. Second, it allows the opportunity to compare the two dimensions with each other, revealing whether employees’ experience of TAW dominates explorative activities over exploitative activities. Although TAW is linked to each individual dimension of EA, it was expected that a similar pattern of linkage to that mentioned in H2 would be found that TAW would be positively linked to each of the two dimensions that constitute EA. The fourth and final hypothesis, therefore, suggests the following:

#### Hypothesis 4:

TAW is positively related to (4a) Employee exploitation and (4b) Employee exploration.

## Methods

### Data collection procedure

This study aims to examine the links between health professional´s TAW and two potential outcomes: IIB and EA. As various psychological states have been found to influence health professionals´ IIB [41], there is still a call for further knowledge and understanding on cultivating homecare professionals´ psychological state [13]. Therefore, this study has focused explicitly on empirically exploring a specific group of health professionals, namely homecare professionals´ TAW, in health services by sampling homecare professionals situated in nine different homecare service institutions in Inland Norway. Previous studies have argued that empirical studies addressing homecare and patient care need to be furthered [42]. In line with this, this study engages homecare professionals who are key in meeting patients’ health needs at homecare. In line with Grasmo et al. [43], this study defines homecare professionals in homecare services as those individuals “with higher university education … or have certifications for more health aides” [43].

Initial and all contact with the nine homecare service institutions was achieved through the health organizations´ Director of Knowledge and Development (DKD). Although this resulted in no direct contact with homecare professionals, it was important to minimize possible influence on the respondents´ answers [44]. This way of communication had to be carried out in accordance, as the Norwegian homecare service sector is under the Data Protection Office, also known as an Internal Hospital Committee, that approves or disapproves whom the homecare professionals can participate in any given study. As such, authors were restricted in direct communication with the homecare professionals. Still, with the help of DKD, all information about the purpose of the study, the questionnaire, the purpose of the study, voluntary participation, anonymity, and consent was forwarded to the health department heads, who then would forward it to their employees. Employees were selected and decided on by DKD and the homecare administration. However, in the invitation to the study, the authors made it known that all homecare professionals had equal opportunity to participate in the study if approved by the DKD and homecare administration. The Norwegian Agency further approved the study for Shared Services in Education and Research (SIKT).

Prior to the gathering of data and after approval from SIKT, a pre-test was completed with the help of a selected group of homecare professionals, including three experts in the field. They reviewed both the claims and quality-checked the back-to-back translation of the claims from English to Norwegian. Through quality checks and pre-tests, we were able to revise our work to avoid leading claims and questions, as well as offer indirect questions and claims that reflected other parties themselves. Upon completing the pre-test and revisions, the final questionnaire was distributed through a link sent to DKD, who furthered it to department heads, who in turn invited their employees to participate. Note that a similar procedure was completed to send a reminder for participation. Participants accessed the survey through Nettskjema (www.nettskjema.no), a platform that provides unique services such as the automatic deletion of IP-addresses upon completion of the survey. As such, and with the approval from SIKT, the study could ensure complete anonymity while ensuring consent through participants ticking a box and “next.” It is important to note that the study employed a self-administered survey where participants had the freedom to complete the survey at their own time and pace.

Around 500 homecare professionals received the survey link, and 258 completed the survey, yielding a 61.6% response rate. Table 1 summarizes the personal characteristics of the 258 participants of this study. Note that in Table 1, this study categorized and grouped Staff roles into three parts: Nurses, Home care professionals, and Others. This is due to the feedback from the DKD and homecare administration, where staff role was originally grouped into administrative work staff, nurses (including registered nurses, nurse practitioners, licensed practical/vocational nurses), social workers, practical nurses, healthcare professionals, other health employees (includes assistive care providers, personal care aides, home health aides, skilled care providers, therapist), homecare professionals with higher education and unskilled. The participants were asked to choose the categories they belonged to accordingly. As the variations between the groups were minor in numbers, also reflected in the overall sample size of 258 participants, and according to the feedback from DKD and homecare administration, the authors grouped the Staff role into three categories as shown in Table 1, where category *nurse* included registered nurse and licensed practical/vocational nurse. The category *homecare professionals included* administrative work staff and healthcare professionals, while the category *other* applied homecare employees unaccounted for in the categories mentioned above, such as homecare professionals with higher education, other homecare professionals, and unskilled homecare employees.

**Table 1:** Personal characteristics of the study sample (*N* = 258)

		%
Staff role	Nurse	34.5
	Homecare professional	49.2
	Other (homecare professionals (bachelor), unskilled)	16.3
Employed	< 5 years	35.7
	6–15 years	26.7
	16–25 years	23.3
	> 25 years	14.3
Work hours	Part-time	66.7
	Full time	33.3
Age	< 35 years	27.5
	35–50 years	38.4
	> 50 years	34.1

### Instruments

Recent research argues for the significance of identifying various factors that are crucial for health professionals in the homecare, factors that can influence their psychological state in their work environments [14, 41, 43]. In line with this, this study furthers knowledge by empirically examining the relationships between health professional´s TAW and the two potential outcomes: IIB and EA. Though the scarcity of previous research on the proposed conceptual model of this study exists, the factors TAW, IIB, and EA all have well-established scales. Therefore, this study could, with ease, use existing scales and fit them to the study´s context: homecare services. Also, as already mentioned, the study performed back-to-back translation on all the claims for two reasons. First, to accommodate to the Norwegian homecare professionals in terms of language. Second, as participants in this study were primarily homecare professionals working within the geographical boundary of Norway, adoption of the claims to the Norwegian context was vital. Therefore, claims of TAW, IIB, and EA were adapted accordingly. Each claim of TAW, IIB, and EA was measured on a seven-point Likert scale, ranging from “strongly disagree” (1) to “strongly agree” (7). In addition, and as already mentioned, the study included demographic characteristics, summarized in Table 1.

*Thriving at work (TAW).* TAW was adapted and measured through a four-item scale from Spreitzer et al. [2]. *Individual innovative behavior (IIB).* IIB was adapted and measured through a 5-item scale from Janssen [45] and Scott and Bruce [46]. *Employee ambidexterity (EA).* EA was adapted and measured through a 6-item scale from Mom et al. [31]. It is important to note that the EA in this study is a second-order factor. Therefore, this study adopted a 3-item scale measuring employee exploration and a 3-item scale measuring employee exploitation, all six items adopted from Mom et al. [31]. This study has termed these both as EA in accordance with previous research and recommendations of Zacher et al. [47]. A full list of the claims used in this study and their construct are summarized in Table 2. Note that the claims used in this study are a part of a larger research project, where the complete questionnaire is used in parts in previous studies of Slåtten et al. [48] and Mutonyi et al. [49].

**Table 2:** Constructs and claims used in the study

Construct	Claims label	Claims
TAW	TAW1	I am continually learning something new in my job
	TAW2	I am continually learning something that makes me better in my job
	TAW3	Generally speaking, I look forward to going to work
	TAW4	I consider my job meaningful
EXPLOR	EXPLOR1	Searching for new possibilities with respect to my work
	EXPLOR2	Focusing on strong renewal of services or working processes
	EXPLOR3	Activities requiring me to learn new skills or knowledge
EXPLOIT	EXPLOIT1	Activities in which I have accumulated a lot of experience
	EXPLOIT2	Activities that I clearly know how to conduct
	EXPLOIT3	Activities I can properly conduct using my existing knowledge
EA (2. order)	EXPLOR	Employee exploration
	EXPLOIT	Employee exploitation
IIB	IIB1	Create new ideas to solve problems
	IIB2	Search out new working methods to improve my job performance
	IIB3	Investigate and find ways to implement my ideas
	IIB4	Promote my ideas so others might use them in their work
	IIB5	Try out new ideas in my work

### Data analysis

Partial least-squares structural equation modeling (PLS-SEM) was used to estimate the conceptual model and test the hypotheses using SmartPLS 3 software [50]. PLS–SEM follows a two-step approach. We confirmed the measurement models by statistical testing (in our case, consisting of only reflective measures) before testing the structural models. EA was specified as a reflective–reflective second-order construct [51]. Based on the PLS-SEM results, mediator effects were estimated and analyzed using the bootstrapping test of Zhao et al. [52].

## Results

### Measurement models

The reflective measurement models were assessed for: (1) internal consistency reliability (the magnitudes of the intercorrelations of the observed variables); (2) convergent validity (the extent to which a variable is positively correlated with alternative variables used to measure the same construct); and (3) discriminant validity (the extent to which a construct is distinct from other constructs). As a minimum standard of measurement quality on the validity and reliability, and as shown in Table 3, we used the ‘rule of thumb’ criteria by Hair et al. [53, 54]. The results set out in Table 3 indicate that we have reliable and valid measurement models.

**Table 3:** Results of the measurement model for the TAW, EXPLOR, EXPLOIT, EA, and IIA constructs

		Convergent validity		Internal consistency reliability		Discriminant validity
Construct	Claims label	Indicator reliability	AVE^a^	Composite reliability	Cronbach’s alpha	HTMT criterion^a^
‘Rule of thumb’		Loading > 0.7	> 0.5	0.7–0.95	0.7–0.95	HTMT interval does not include 1
TAW	TAW1	0.86	0.67	0.89	0.84	Yes
	TAW2	0.88				
	TAW3	0.77				
	TAW4	0.77				
EXPLOR	EXPLOR1	0.89	0.79	0.79	0.87	Yes
	EXPLOR2	0.88				
	EXPLOR3	0.89				
EXPLOIT	EXPLOIT1	0.92	0.86	0.92	0.92	Yes
	EXPLOIT2	0.94				
	EXPLOIT3	0.92				
EA (2. order)^b^	EXPLOR	0.91	0.85	0.92	0.80	Yes
	EXPLOIT	0.93				
IIB	IIB1	0.78	0.67	0.91	0.87	Yes
	IIB2	0.83				
	IIB3	0.82				
	IIB4	0.81				
	IIB5	0.83				

^a^*AVE* Average variance extracted, *HTMT* Heterotrait–monotrait ratio of correlations

^b^The validation criteria for the second-order construct is based on the guidelines by Sarstedt et al. [49]

### Structural models

**Fig. 2:**
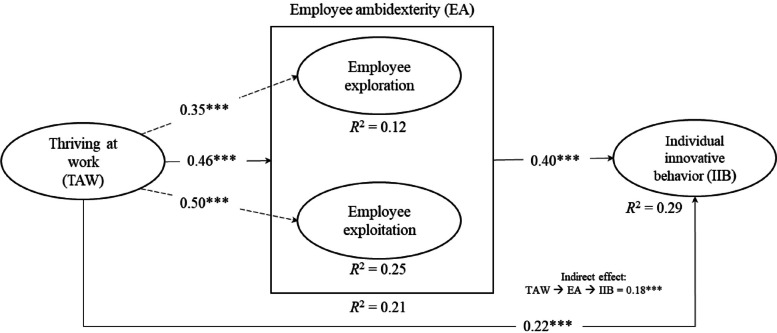
Results from the structural model of the links between TAW, EA, and IIB. Standardized coefficients (*** < 0.01)

To test the hypotheses, two structural models were estimated. To investigate hypotheses H1–H3, we specified and estimated Model 1, with TAW specified as the exogeneous construct, IIB as the endogenous construct, and EA as the mediator variable construct. To test hypotheses H4a and H4b, in Model 2, TAW was specified as the exogeneous construct and employee exploration and employee exploitation as endogenous constructs. The results are presented in Fig. 2.

For the endogenous constructs, we examined the in-sample predictive power of the model using *R*2. The *R*2 values were 0.21 for EA, 0.25 for IIB, and 0.25 for employee exploitation. Based on the ‘rules of thumb’ [53, 54] these *R*2 values were considered moderate. For employee exploration, *R*2 was 0.12. All the coefficients were statistically significant at the 0.01 level. The path coefficient between TAW and IIB was positive (*b* = 0.22), supporting H1. H2 was also supported because the relationship between TAW and EA was positive (*b* = 0.46). In addition to the positive direct effect between TAW and IIB, Fig. 2 shows that EA had positive indirect effects (*b* = 0.18) and a complementary mediation effect on the relationship between TAW and IIB, supporting H3. TAW was positively related to both employee exploration (*b* = 0.35) and employee exploitation (*b* = 0.50), supporting H4a and H4b, respectively.

### Additional analysis

The study also includes summary statistics of the variables (see Attachment 1). We also ran additional analysis, specifically bivariate analysis (see Attachment 2), to explore differences in response by type of health worker, age, and experience. The results demonstrated non-significant findings and are, therefore, not discussed further.

## Discussion

TAW is a relatively new concept [55]. It stems from and is rooted within positive psychology, which is the “study [of] what is good in life” [56]. As shown in this study, when employees experience a “good life” at work, as reflected in their TAW, the experience is associated with important outcomes related to their work role.

The study offers three contributions. First, it extends and deepens previous research on TAW among homecare professionals, which addresses a previously substantial knowledge gap. Second, it contributes to our understanding of how TAW is related to outcomes in this study referring to EA and IIB. In doing so, it follows the suggestion of Kleine et al., who stated: “future research should examine the relationships between thriving and … its … outcomes” [6]. Third, the study deepens our insight into activity processes, reflected in EA, and how these processes are linked to both TAW and IIB. The study responds to a call for more research both on TAW [14] and on the potential processes linked to TAW [5]. Thus, it contributes to a relatively neglected area within health services research.

According to Riaz et al., “individuals [*sic*] feeling of thriving at work affect [*sic*] their behavior” [57]. The findings from this study support this statement. TAW was found to be positively linked to the IIB of employees. Previous research has also found support for a positive association between TAW and IIB. For example, Riaz et al.’s study of 412 employees in a global investment company found a significant association between TAW and innovative work behavior [57]. In a similar vein, Usman et al.’s study of 201 employees in the telecom sector of Pakistan [28] found a positive relationship between TAW and innovative work behavior. Other studies also support the findings from this study, although, to the best of the authors´ knowledge, this study is a pioneering research examining the proposed relationships among health professionals within the health services context. Consequently, our findings extend the body of previous research on TAW within health services research that has previously been dominated by examining only the antecedents to TAW (e.g., [7–12]) or only conceptually suggesting TAW to be associated with specific outcomes.

To our knowledge, only Moloney et al.’s study [13] has linked TAW to specific outcomes. In their integrative literature review of papers from 2005–2019, the authors suggest the sustainability of human resources and better health and well-being as two potential outcomes of homecare professionals experiencing TAW. Clearly, both are relevant and important outcomes of TAW. However, our paper extends the study of Moloney et al. [13] in two ways. First, it links IIB as a new outcome of homecare professionals’ TAW. Second, it both conceptualizes and empirically validates the association of IIB with the homecare professionals’ experience of TAW. By doing so, it provides a solid foundation to conclude that TAW is a driving force for IIB among homecare professionals, with the two factors embraced in the concept of TAW (vitality and learning) capable of motivating and positively fueling employees’ engagement in IIB.

This study also contributes to the extension of previous research on innovation in health services research. The importance of innovation in healthcare organizations (as well as in other organizations) for improving quality, productivity, and service offerings is well known. Länsisalmi et al. describe innovation as a “critical capability of all healthcare organizations” [58].

A majority of previous studies on innovation in health care have been dominated by organizational or group-level perspectives or a focus on the adoption of innovation [51]. Consequently, only a small number of studies have centered on studying innovation at an individual level [58], which was the aim and focus in this study. However, in recent years, there has been an increase in the number of studies taking an individual-level perspective on innovation. Examples of factors linked to IIB within health services research include employees’ experience of empowerment [59], motivation, the experience of stress [60], creativity, psychological capital, and autonomy support [14], organizational culture [41], and organizational vision integration and organizational attractiveness [61]. Clearly, each of these studies contributes to extending our understanding of factors that drive IIB. However, none of these has examined the linkage between TAW, EA, and IIB among health employees. Thus, our study extends our insight into factors that promote IIB in a specific work role. It is interesting to note that TAW and EA explain about 30% (*R*2 = 0.29) of the variance in IIB. Clearly, these two factors have substantial capability to drive employees’ innovative behavior. Thus, findings from this study support Kim and Park’s statement that “individuals are the primary agents to develop and execute innovative ideas” [62].

The findings of this study go beyond contributing to knowledge of how TAW and EA are each linked to IIB. Specifically, EA was suggested as a process variable or mediator between TAW and IIB. The motivation for choosing EA was based on a recent study by Pertusa-Ortega et al. [34]. In their literature review, the authors urged researchers to place more focus on ambidexterity at an individual level [34]. Based on an adjustment of March’s [38] ideas to address the health professional sample of this study, EA was referred to as the combination of two contradictory activities: exploration activities and exploitation activities of employees. The activities embraced in EA are about combining the search for novel knowledge and experiences while simultaneously seeking to deepen one’s existing knowledge and experiences to potentially benefit one’s work role. As revealed by this study, EA is associated with both TAW and IIB, thus indicating that EA operates as a mediator. More precisely, using the terminology of Zhao et al. [52], EA has a “complementary mediation” effect on the relationship between TAW and IIB. Consequently, two (complementary) “routes” leading from TAW to IIB have been identified. The first is a direct route from TAW to IIB, and the second is an indirect route in which EA functions as a mediator. Thus, the study reveals some of the complexity that explains how the linkage between TAW and IIB might operate. To the best of the authors´ knowledge, the pioneering findings of this study, within (but also outside) health services research, advance knowledge by conceptualizing and empirically testing these linkages.

The findings from this study are also interesting when inspecting the individual outcome of EA on IIB. According to Luu et al., there has been lack of research related to “customer outcomes of individual ambidextrous behavior” [63]. In a similar vein, Schnellbächer et al., recently noted that “… few studies have assessed the performance effects of individual ambidexterity” [64], or what this study labeled as EA. In this study, the “customer” outcome of EA is manifested in the IIB of employees. Consequently, this study responds to Luu et al.’s [63] and Schnellbächer et al.´s [64] call for more research within this area. The strength of the positive direct link between EA and IIB was found to be *b* = 0.40. This finding is especially interesting when compared with the positive direct effects of TAW on IIB, which were found to be *b* = 0.22. The comparison clearly supports the importance of EA for employees’ innovative behavior. It provides support that, independent of whether IIB is incremental or radical, it is the combination of the two forms of behavior that is embraced in EA. Consequently, EA serves as a necessary input to IIB.

The findings reveal that EA is a significant driver for IIB compared to TAW. Although scarce previous research has examined the same linkage, the findings provide some support for a study by Luu et al. [63] in which the authors examined, among several other factors, the links between a concept labeled “frontline public employees’ individual ambidexterity” (a concept comparable to EA in this study) and a concept labeled “customer value co-creation.” Customer value co-creation was defined as the actual engagement of the customer in value co-creation. Luu et al. [63] found support for a link between employees’ individual ambidexterity (or EA) and customer willingness to engage in value co-creation. Although there are clearly differences between our study and the study of Luu et al. [63], our study provides some support for a positive linkage between EA and outcomes related to customers, as reflected in the concept of IIB. However, research should explore this in more detail. One suggestion would be to study the experience of IIB, stemming from EA, from a “receiver” or customer perspective. This would provide more objective information on both customer perception of the degree of innovativeness (IIB) as well as on the customer perception of the quality of the innovation (IIB) offered. The latter is essential because it is reasonable to assume that not all types of innovation are recognized as useful and “good” from a receiver’s perspective.

### Practical implications

In the literature, workplace thriving is described as a personal resource [28] and defined as a state [2]. This study's results provide two good reasons why managers of health organizations should focus on and continuously cultivate the personal resources embraced by individual employees’ TAW.

First, TAW is capable of having an impact on EA. Of all the linkages examined in this study, TAW was found to be most associated with EA (*b* = 0.46). This is an intriguing finding. According to Schnellbächer et al., there is a lack of “deeper understanding of how individual ambidexterity [or EA] may be nurtured” [64]. To our knowledge, this is a pioneering study in health services research revealing TAW as a significant enabling factor for EA. This implies that the more health managers strengthen TAW, the more homecare professionals will be motivated to engage in EA. Further, when inspecting the single linkages between TAW and the two dimensions of EA, the findings for hypotheses 4a and 4b reveal that TAW is capable of nurturing both employee exploitation (*b* = 0.50) and employee exploration (*b* = 0.46).

Second, as discussed previously, TAW is able to stimulate and motivate IIB both directly and indirectly through EA as a mediator. Consequently, TAW functions as a potential universal management “tool.” When managers have a determined focus, they have the opportunity, through TAW, to simultaneously achieve multiple desirable outcomes (EA and IIB).

Consistent with the important findings from this study, Goh et al. recently commented that “thriving at work has emerged as a critical psychological driver of individual growth and development and is essential for … performance” [38]. Consequently, based on the importance of thriving revealed by this study, all managers of homecare services and homecare professionals should do their best to comply with the following slogan manifested in their daily management practices: *“Strive to let them thrive!”.*

### Limitations and future research

Because TAW is a relatively new concept and scant research has been undertaken on it, there are several opportunities to continue exploring this “black box” within health services research. First, TAW was found to be linked to two outcomes, EA and IIB, respectively. A noteworthy finding is identifying a positive linkage between TAW, EA, and IIB. However, future research should try to extend the list of outcomes. Several factors could potentially be included. One example is service quality of care which, in a target group of homecare professionals who work directly with “customers,” should be prioritized in future research. There are two alternative strategies to capture information about the perception of service quality of care. The first is to measure service quality of care from an employee perspective, which can be labeled as employee-perceived service quality. This is a subjective way to measure service quality of care. The idea behind this strategy is that employees, based on their self-evaluation, can evaluate whether their service quality of care will be perceived as “good” or “bad” in the eyes of their customers. Although self-evaluation is an often-used strategy, it only provides an indication of the level of “goodness” of service quality of care offered. Therefore, the second strategy—asking “customers” directly about their perception of service quality of care offered from individual homecare professionals—would be preferred as a more objective method. Regardless of the strategy chosen, there is an urgent need to include service quality as a potential output of TAW in future research.

Second, in continuation of the aforementioned suggestion, future research should also study the linkage between service quality of care, the IIB of employees, and their experience of TAW. Innovation is proposed to increase the quality of health care [65]. In this reasoning, it is assumed that IIB functions as a mediator in the relationship between TAW and service quality of care. Although there are several reasons to conceptually expect a linkage between the three concepts, no previous study within health services research has specifically examined this empirically. An extension would be adding IIB as a mediator and including creativity in the equation. Previous research has linked employee creativity to IIB [14]. Creativity can be seen as a necessary precondition to IIB. Therefore, it would be useful to understand whether TAW is linked to employees’ individual creativity (cognitive creative thinking skills) and IIB and how these may be linked to service quality of care. Such an approach would contribute to new insights and knowledge about the importance of TAW and simultaneously provide practical implications for managers of health organizations.

Third, based on the identified knowledge gaps in previous health services research, this study limited its focus to the potential outcomes of TAW, and did not attempt to identify potential antecedents for TAW. Although few studies within health care have taken the latter approach, there is clearly a substantial need for an examination of the factors that potentially promote TAW.

Spreitzer et al., who first proposed TAW, considered that “thriving at work is socially embedded” [2]. There are several factors that could be relevant under the umbrella term “socially embedded” and thus qualify as factors that promote TAW for individual employees’ experiences of TAW. In recent reviews of the TAW literature (e.g., [6, 38]), one will find various factors promoting TAW that could potentially be adopted when studying homecare professionals in specific work roles. In reference to our study, one suggestion would be to link ambidextrous leadership to TAW in future research.

It is well known that leaders in organizations have significant potential to stimulate, motivate, and form employees in multiple ways, both through their formal roles and how they perform their leadership practices. According to Rosing et al., ambidextrous leadership refers to a leader’s “ability to foster both explorative and exploitative behaviors in followers by increasing or reducing variance in their behavior and flexible switching between those behaviors” [66]. It is reasonable to assume that when leaders appropriately combine the two ways of fostering behavior they will promote both employees’ affective (vitality) and cognitive (learning) aspects of TAW in a positive way. Stated another way, with reference to the previous discussion of TAW, ambidextrous leadership can trigger both the hedonic and eudaemonic qualities combined in a person’s experience of TAW. Thus, there are good reasons to assume a positive linkage between ambidextrous leadership and TAW. To our knowledge, only one previous study has examined the link between ambidextrous leadership and TAW. Specifically, Usman et al. [28] found ambidextrous leadership and TAW to be significantly related (*b* = 0.415) in a sample recruited from the telecommunications sector. Interestingly, they concluded that ambidextrous leadership would “contribute to the boosting levels of thriving (i.e., vitality and learning)” [28]. Their study also found support for a link between ambidextrous leadership and innovative work behavior. However, although Usman et al.’s study [28] is undoubtedly valuable, their participants were not drawn from the healthcare sector. Consequently, future research should reveal whether a similar positive linkage exists between ambidextrous leadership and TAW among homecare professionals. For example, further exploration into the differences between factors that motivate various types of homecare professionals to uncover the role of TAW in promoting EA and IB would be an exciting area to focus on.

In addition, there is also an opportunity to examine how ambidextrous leadership is, either directly or indirectly, related to the other variables we have previously suggested—service quality of care, IIB, and employee creativity—but which have not yet been examined in health services research. Including one or several of these concepts would contribute to further exploring the “black box” and extend our understanding of the factors related to the work role of homecare professionals.

Fourth, EA was found to be directly linked to both TAW and IIB while simultaneously functioning as a mediator between them. On the basis of this promising result and the multiple roles EA seems to have, this concept should be explored in future research. As previously mentioned in this paper, there is a lack of studies focusing on ambidexterity at an individual level [30, 33–35]. To our knowledge, this is a pioneering study within health services research to examine EA at an individual level among homecare professionals. We therefore strongly recommend that EA should be studied in relation to both potential antecedents and effects. There are a range of opportunities for this. One suggestion is to link EA to the aforementioned concept of ambidextrous leadership. Previous studies, although undertaken outside health services research, have revealed ambidextrous leadership to be positively linked to employee exploration and exploitation (EA) and important outcomes such as innovative performance [47] and customer value co-creation [63]. Future research should test whether EA shows a similar pattern among employees working as homecare professionals. It would also be possible to connect EA to a range of factors that promote TAW. For example, EA could be linked to contextual factors that promote TAW manifested in concepts such as organizational culture and climate, relationship learning, organizational attractiveness, or/and different types of support (e.g., from leaders, coworkers or teams).

Moreover, it would also be possible to link EA to more individual-related factors that promote TAW, such as employees’ individual learning orientation, psychological capital, personality traits, self-efficacy, TAW, work engagement, emotions, and so forth. When considering the outcomes of EA, there are also several opportunities. Future research could link EA to outcomes relevant to both homecare professionals, such as task performance, commitment, service quality of care, creativity, creative performance, turnover intentions, and commitment to change. A further and interesting outcome to include would be that of “quality-productivity” ambidexterity to determine whether, how, and to what extent EA helps homecare professionals to achieve the duality (or ambidexterity) of offering a high level of service quality of care while simultaneously achieving a high level of productivity in their work role. Because EA is a relatively new concept, especially within the domain of health services research, there are several opportunities to extend our insight and knowledge about it.

Fifth, in this study, TAW is conceptualized and defined as an employee’s psychological state composed of the joint experience of vitality and learning [2]. This definition clearly focuses on the individual employees and potential variation in the (dynamic) state of TAW of individuals. Although this clearly is a valuable and appropriate way to define and study TAW, other approaches for how to study and conceptualize TAW could be considered in future research. In a recent review by Goh et al. [38], the authors mention two other types of conceptualizations of thriving—dyadic thriving and collective thriving—in addition to individual thriving. The common characteristics of these two other types of conceptualizations are a change from focusing only on the individual to studying how thriving is manifested among several individuals. In health care, dyadic thriving refers to variations in levels of thriving where two individual homecare professionals are working together or in cooperation. Collective thriving refers to variation in levels of thriving where several (more than two) individual homecare professionals are working together or in cooperation, such as in a team, department, or organization. It is easy to understand the value and potential practical implications of studying TAW from either a dyadic or collective perspective (or both) within a healthcare context. However, research to date on dyadic and collective thriving constitutes an “un-ploughed area” within health services research. Consequently, this focus on TAW is added to other suggestions in this section for future research considerations within the domain of health services research. Following these suggestions will provide more answers and validation for the proposed management practice slogan: *“Strive to let them thrive!”.*

Sixth, this study is limited by its cross-sectional design, self-selection bias, social desirability bias, the possibility of reversal of causality in relationships, and its small sample (258 respondents). Specifically, the empirical data in this study were collected at one point in time. Homecare professionals across nine municipalities are included in this study, but the municipalities chosen for this study are all concentrated in a fixed geographical location in Norway. Also, the study found no significant findings when running a simple bivariate analysis to explore possible differences in response by a selected control variable. This might be attributed to the small sample size of this study, although this might not be the case in future studies. In addition, the study focused on a sample of 258 homecare professionals, implications for opportunities for generalizing the findings to healthcare workers. Consequently, the results of this study are not generalizable to other health organizations, nor to a broader population. Also, this study limited its self-selection bias; however, this might still occur. Therefore, the results of this study should be interpreted carefully. Moreover, although this study followed the recommendations of McCrae and Costa [67] in reducing social desirability bias (SDB) through anonymity, wording of claims, self-administered questionnaire, and indirect questioning, to name a few, the study might still suffer from SDB because the DKD handled all communication with the homecare organizations and their employees. As such, the results of this study can serve as a stepping-stone to future research in including various health organizations, testing causal and reversed casual relationships, and utilizing other means of data collection, such as, though not limited to, qualitative studies and longitudinal analysis. This was done to minimize method bias. In addition, the limitations linked to online surveys are known to include self-selection and shared response bias, owing to the nature of self-report measures. Consequently, as suggested by Hair et al. [44], these limitations offer opportunities that the contributions of future studies could benefit from.

## Conclusion

This study contributes to our insight into potential outcomes of homecare professionals’ TAW, which has been a neglected area within the domain of health services research. The findings reveal that TAW is a significant driver of both EA and IIB. Consequently, managers should be aware of changes in the level of TAW in their individual employees. A practical implication is the importance of continuously cultivating the TAW of individual employees and revealing whether they experience a sense of being energized and feeling alive as members of the organization. Managers should, therefore, be determined to strive to let all of their employees thrive.

## Supplementary Information

Supplementary Material 1.

## Data Availability

The data sets used and/or analyzed in this study are available from the corresponding author on reasonable request.

## Abbreviations

PLS-SEM: Partial least structural equation modeling
AVE: Average variance extracted
HTMT: Heterotrait–Monotrait Ratio of Correlations
SIKT: Norwegian Agency for Shared Services in Education and Research
DKD: Director of Knowledge and Development
TAW: Thriving at work
IIB: Individual innovative behavior
EA: Employee ambidexterity
EXPLOR: Employee exploration
EXPLOIT: Employee exploitation
