# Supplementary material for: Why should we strive to let them thrive? Exploring the links between homecare professionals thriving at work, employee ambidexterity, and innovative behavior
Source: BMC Health Serv Res. 2025 Jan 27;25:154. doi: 10.1186/s12913-025-12293-9 (PMC11773897; doi:10.1186/s12913-025-12293-9)
Supplement: Supplementary file 1 — Supplementary Material 1. [file 12913_2025_12293_MOESM1_ESM.docx]

**Attachment 1** Summary of the variables included in this study.

| Construct | Claims label | Mean | Standard deviation | Minimum | Maximum |
| --- | --- | --- | --- | --- | --- |
| TAW |  |  |  |  |  |
|  | TAW1 | 5.85 | 1.14 | 1 | 7 |
|  | TAW2 | 5.90 | 0.99 | 1 | 7 |
|  | TAW3 | 5.33 | 1.53 | 1 | 7 |
|  | TAW4 | 6.33 | 0.98 | 1 | 7 |
| EXPLOR |  |  |  |  |  |
|  | EXPLOR1 | 5.85 | 0.92 | 1 | 7 |
|  | EXPLOR2 | 5.81 | 0.98 | 1 | 7 |
|  | EXPLOR3 | 6.03 | 0.96 | 1 | 7 |
| EXPLOIT |  |  |  |  |  |
|  | EXPLOIT1 | 6.08 | 0.92 | 1 | 7 |
|  | EXPLOIT2 | 6.13 | 0.93 | 1 | 7 |
|  | EXPLOIT3 | 6.13 | 0.91 | 1 | 7 |
| IIB |  |  |  |  |  |
|  | IIB1 | 5.85 | 0.87 | 3 | 7 |
|  | IIB2 | 5.72 | 0.89 | 3 | 7 |
|  | IIB3 | 5.31 | 1.05 | 1 | 7 |
|  | IIB4 | 5.43 | 1.06 | 1 | 7 |
|  | IIB5 | 5.41 | 1.03 | 1 | 7 |
| *Note: TAW: Thriving at work; EXPLOR: Employee exploration; EXPLOIT: Employee exploitation; IIB: Individual innovative behavior. N = 258.* | | | | | |

**Attachment 2** Results of the bivariate analysis to explore differences in response by type of health worker, age, and experience. *N = 258.*

| IIB | Coefficient | Std. err. | t | P>t | [95% conf. | interval] |
| --- | --- | --- | --- | --- | --- | --- |
|  |  |  |  |  |  |  |
| Age | .0055412 | .0062229 | 0.89 | 0.374 | -.0067117 | .017794 |
| Type of health worker | .263562 | .3236323 | 0.81 | 0.416 | -.3736669 | .9007909 |
| Experience | -.0461289 | .0736457 | -0.63 | 0.532 | -.1911366 | .0988787 |
| _cons | -.1522223 | .2221497 | -0.69 | 0.494 | -.5896329 | .2851883 |
